# Genetic diversity and community composition of arbuscular mycorrhizal fungi associated with root and rhizosphere soil of the pioneer plant *Pueraria phaseoloides*

**DOI:** 10.1002/imt2.51

**Published:** 2022-09-30

**Authors:** Yaqin Guo, Qicheng Bei, Beloved Mensah Dzomeku, Konrad Martin, Frank Rasche

**Affiliations:** ^1^ Department of Agronomy in the Tropics and Subtropics, Institute of Agricultural Sciences in the Tropics (Hans‐Ruthenberg‐Institute) University of Hohenheim Stuttgart Germany; ^2^ Department of Soil Ecology Helmholtz Center for Environmental Research ‐ UFZ Leipzig Germany; ^3^ Department of Horticulture CSIR ‐ Crops Research Institute Kumasi Ghana

## Abstract

The pioneering plant *Pueraria phaseoloides* had a strong modulation effect on arbuscular mycorrhizal fungi (AMF) communities. Irrespective of geographical location, community composition of AMF in rhizosphere soil differed from that of the root. Co‐occurrence network analysis revealed two AMF keystone species in rhizosphere soil (*Acaulospora*) and roots (*Rhizophagus*) of *P. phaseoloides*.

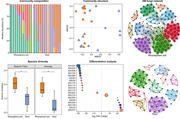

## INTRODUCTION

Arbuscular mycorrhizal fungi (AMF) ensure the survival of plants by facilitating access to limited resources, particularly in degraded ecosystems [1], thereby playing a crucial role in sustaining ecosystem processes and functions [2, 3, 4]. The composition, diversity, and abundance of AMF communities depend on several factors. For instance, meta‐analysis revealed that AMF exhibit biogeographic patterns at a global scale [5]. At a local scale, both soil and biogeographical factors were shown to determine AMF communities [6]. Numerous studies have proven that soil conditions also determine AMF diversity and composition (Supporting Information: Table S1). However, AMF have an idiosyncratic response to soil conditions, and there is no consensus on their relative importance [7]. Several studies showed that soil pH is an important factor to determine AMF communities [8, 9]. It was also reported that a higher level of phosphorus (P) limited the diversity of AMF [10]; but another study showed that soil texture, rather than pH or P, affects AMF composition in an agriculture soil [11]. This divergence may be the result of having targeted different ecosystems, host plants and sample types (Supporting Information: Table S1). More importantly, plants exert strong effects on the diversity and composition of their asscociated AMF [12, 13], but the specificity of the association between plant host and AMF taxa is generally low [14]. In addition, the same plant species may reveal differences in AMF communities between plant compartments, that is, root and rhizosphere soil [15, 16, 17, 18].

AMF have been classified into different functional groups based on biomass allocation, that is, rhizophilic guild and edaphophilic guild [19]. The rhizophilic guild is thought to allocate more arbuscular mycorrhizal (AM) biomass to roots than soil, such as *Rhizophagus*; while the edaphophilic guild is thought to allocate more AM biomass to soil than roots, like *Acaulospora*. However, caution must be taken when classifying AMF families into guilds, due to the different technologies used [20]. In addition, plants could affect AMF richness by delivering more carbon to beneficial symbionts which could facilitate the competition with others [21, 22]. Especially in degraded ecosystems, “founder AMF” species might benefit from plant‐derived carbon to colonize the plant roots, thus would outcompete “AMF latecomers”, thereby leading to differences between root and rhizosphere soil [23]. Thus, understanding the extent to which various factors modulate abundance, diversity, and selective root‐colonization of AMF (i.e., niche differentiation) is essential not only for maintenance of ecological processes in agroecosystems [24, 25], but also for facilitating the restoration of degraded ecosystems [26, 27, 28]. AMF has been verified as pioneering microorganisms in sand dunes [29], river floodplains [30], and volcanic areas [31]. Although the role of AMF has been studied intensively in different ecosystems with different plant species (Supporting Information: Table S1), AMF diversity and communities in association with pioneer plants in heavily degraded ecosystems remain largely unexplored. These include post‐mining sites [32], which are frequently found in West Africa. This is especially true for Ghana, which is the largest producer of gold in Africa, and the sixth largest producer in the world [33]. With a rate of 2600 hectares per year, natural vegetation in Ghana has been intensively converted into gold mining sites [34]. After surface mining, the land is usually left abandoned and free of vegetation, offering space for colonization by pioneer plants. In Ghana, *Pueraria phaseoloides* (Roxb.) Benth. (tropical kudzu), a perennial, N_2_‐fixing legume, has been found as a pioneer plant species that colonizes vigorously post‐mining sites (Y. Guo, personal communication, 2019). This was similarly observed in Indonesia [35]. It could be speculated that such successful colonization and potential adaptation to various site conditions is reinforced by the symbiosis with AMF. This assumption is rationalized by an earlier study, revealing that *P. phaseoloides* failed to establish without the presence of symbiotic AMF [36].

In view of the ecological advantage of *P. phaseoloides* to colonize efficiently post‐mining sites, it is imperative to disentangle the factors shaping the mycorrhizal communities associated with *P. phaseoloides*, considering benefits for degraded land restoration. Hence, we investigated the genetic diversity and composition of AMF communities in the rhizosphere and roots of *P. phaseolides* growing under the prevailing soil conditions at abandoned, highly disturbed post‐mining sites in Ghana. High throughput DNA sequencing was employed to analyze AMF communities in degraded mining soils. Different from morphological methods, which rely mainly on spore identification, DNA‐based approaches capture genetic information from hyphae, mycorrhizal roots and spores [37]. We hypothesized that, due to the strong adaptation potential of *P. phaseolides* in different environments, host identity is a major driving factor shaping AMF communities, assumably stronger than local factors related to geography and soil conditions. Secondly, we hypothesized that the high adaptability of *P. phaseolides* may be reflected in the selection of AMF specific species from the rhizosphere soil. By testing this, our main ambition is to fill a gap in the understanding of the ecological status of AMF in degraded mining soils and to provide a scientific basis for developing AMF‐based strategies for restoring degraded lands.

## RESULTS

### Overall sequencing and taxonomic assignments

In total, 2,312,972 raw reads were obtained with 301 bp average read length from Illumina MiSeq® sequencing. The quality control (quality scores > 35) reduced the reads to 2,039,447 with 271 bp sequences on average (i.e., an average of 11.8% of the reads was discarded). Rare amplicon sequence variants (ASV) with a frequency of less than 0.1% of the mean sample depth were removed (see explanation in “Bioinformatic analysis” section). After removal of rare ASV (16.8%), a total of 1,746,146 reads remained (Supporting Information: Table S2).

**Figure 1:**
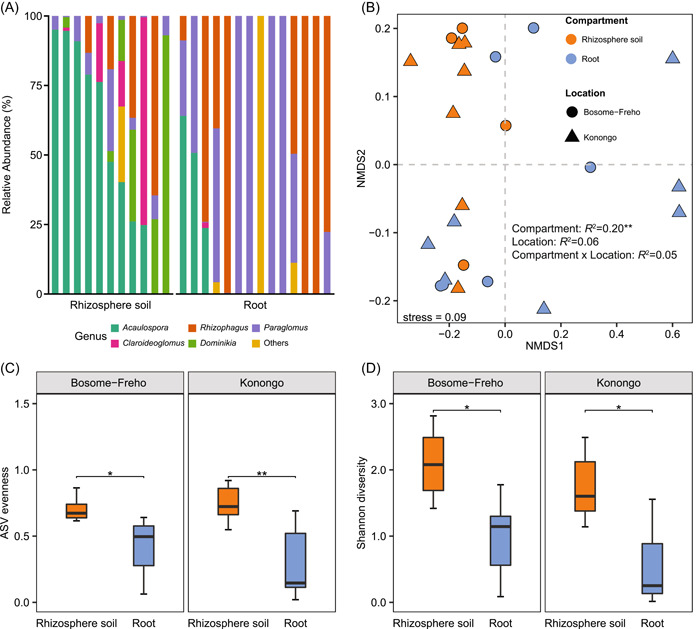
The overall structure of arbuscular mycorrhizal fungi (AMF) communities. (A) Genus distribution of amplicon sequence variants (ASV) using relative abundance of AMF associated with rhizosphere soil and root. Eleven rhizosphere soils and 14 root samples are displayed in separate stacked bars. Different genera are displayed in different colors, and the low abundance genera (<10%) are grouped together (others). Relative abundance of each genus across compartments is displayed in Supporting Information: Figure S2. (B) Nonmetric dimensional scaling (NMDS) analyses of weighted metric distance of AMF communities. (C) and (D) α‐diversity of field samples. (C) Pielou's evenness. (D) Shannon diversity. The boxes represent the range between 75th and 25th quartiles. The line within the box represents the median. The whiskers represent the lowest and highest values extending 1.5 of the interquartile range. NS, nonsignificance. * Significance (*p* < 0.05); ** Significance (*p* < 0.001).

Non‐*Glomeromycota* sequences were filtered according to the NCBI database (see details in “Bioinformatic analysis” section), resulting in 195 ASV to the phylum *Glomeromycota* for downstream analysis. Among them, 102 ASV belonged to the rhizosphere soil and 72 ASV were discovered in roots, while 21 ASV shared both compartments of rhizosphere soil and root (Supporting Information: Figure S1). Phylogenetic analysis assigned 195 ASV to 8 genera: *Acaulospora* (72), *Rhizophagus* (43), *Paraglomus* (43), *Dominikia* (16), *Claroideoglomus* (7), *Funneliformis* (7), *Septoglomus* (4), *Diversispora* (3). *Acaulospora*, *Rhizophagus, Paraglomus*, and *Septoglomus* were found both in the roots and rhizosphere soil. *Dominikia* and *Claroideoglomus* were only detected in the rhizosphere soil, while *Funneliformis* and *Diversispora* were only found in the roots (Figure 1A and Supporting Information: Figure S2).

### Alpha diversity of AMF

Rarefaction curves display the number of sequences, which have reached adequate coverage (saturation) of AMF diversity, as a quality requirement for downstream analysis (Supporting Information: Figure S3). The α‐diversity indices were displayed and statistical differences were annotated for both locations (Konongo, KN; Bosome‐Freho, BF) (Figure 1C,D and Supporting Information: Figure S4). At both locations, more ASV were observed in rhizosphere soil than in roots, although this difference was not significant (*p* > 0.05) (Supporting Information: Figure S4A). At both locations, ASV evenness and diversity of rhizosphere soil were, however, higher than in roots (*p* < 0.05) (Figure 1C,D). ASV richness of rhizosphere soil in BF was higher than in KG (*p* < 0.05), while evenness and diversity of ASV did not reveal any difference between the two locations (data not shown).

### Beta diversity (community composition) of AMF

AMF composition between the two compartments was clearly separated, but not between the locations (Figure 1B). The stress value of NMDS analysis was 0.09, reflecting the significant variation of AMF communities among factors [38]. To verify the significance of NMDS analysis, factors (plant compartment, location) were further examined using permutational multivariate analysis of variance (MANOVA) test (Figure 1B). Results indicate that the plant compartment was a crucial determinant to modulate AMF composition (*p* < 0.05), explaining more than 20% of variation. However, the effect of location on AMF composition was not significant, explaining only 4% of variation (*p* > 0.05).

### Relationship of AMF with soil characteristics

The relationship between AMF richness (observed ASV) and diversity (Shannon diversity) and soil characteristics was analyzed (Supporting Information: Table S3). The contents of soil calcium (Ca) and soil pH had negative correlations with both AMF richness and diversity (*p* < 0.05). The content of soil zinc (Zn) only had a negative correlation with AMF richness (*p* < 0.05). Distance‐based redundancy analysis (db‐RDA) was carried out to examine the influence of soil characteristics on AMF composition. Results indicate that soil characteristics had no effect on AMF composition (*p* > 0.05) (Supporting Information: Table S4).

### Differences in AMF communities between plant compartments

**Figure 2:**
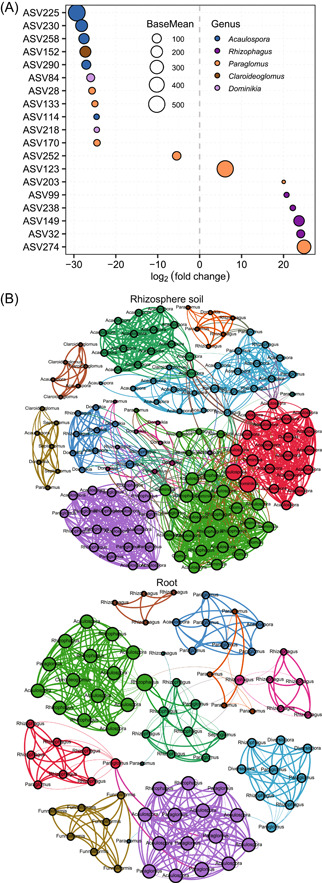
The differences between amplicon sequence variants (ASV) and keystone taxa identification. (A) DeSeq2 plot showing the difference of amplicon sequence variants (ASV) between the two plant compartments. Different colors represent different genera, and different point sizes represent different mean values, after normalization through DeSeq2. (B) Co‐occurrence network in rhizosphere soil and root compartment. Each node represents one ASV labeled by genus. A node was verified by a robust (Spearman's correction coefficient *R* > 0.6) and significant (*p*_FDR_ < 0.05) correlation. The size of each node is relational to the number of connections, while nodes with the same color display the same module. The thickness of each connection between two nodes is relational to the strength of Spearman's correlation coefficient.

With the analysis of DeSeq2, 12 ASV were found to be more abundant in the rhizosphere soil than in root samples, whereas 7 ASV were more abundant in the root compartment than in the rhizosphere soil (Figure 2A). In the root compartment, ASV were affiliated to two genera: *Rhizophagus* (4) and *Paraglomus* (3). In rhizosphere soils, ASV were assigned to *Acaulospora* (5), *Paraglomus* (4), *Dominikia* (2) and *Claroideoglomus* (1). Network of the rhizosphere soil had more nodes and edges (123 nodes, 948 edges) than the root compartment network (86 nodes, 468 edges). The modularity index (rhizosphere soil: 0.78; root: 0.79) was above 0.4, indicating a modular network structure [39]. The average degrees of rhizosphere soil and root were 17.6 and 10.9, respectively, and the average clustering coefficient was 0.84 for rhizosphere soil and 0.95 for root. The detailed results of co‐occurrence networks are listed in Supporting Information: Tables S5 and S6. On basis of the network analysis, ASV230 (*Acaulospora*) and ASV238 (*Rhizophagus*) were determined as keystone taxa for the rhizosphere soil and root compartment, respectively (Figure 2B and Supporting Information: Tables S5 and S6).

## DISCUSSION

Our results showed that the root of the pioneer plant *P. phaseoloides* revealed a lower ASV richness and diversity of AMF than the associated rhizosphere soil, a finding in line with other plant species [15, 16]. This confirms the general view that the rhizosphere soil provides an important reservoir of AMF for plants, from which plants only recruit a proportion at a certain time [40]. More importantly, the plant compartment of *P. phaseoloides* exerts, independent of geographical location or microbiome provenance, a strong effect on microbial consortia shift, indicating a selective preference for associated AMF. It was shown that geographic distance had a small effect on AMF communities, because a single plant species in agricultural land may homogenize the AMF communities over a certain distance [41]. Furthermore, the host compartmentalization of microbial communities facilitates the decoupling from effects of habitat fragmentation [42]. In our study, *P. phaseoloides* plays an important role in shaping AMF communities, thereby overriding geographic factors. However, the symbiosis between plants and AMF is generally considered as nonspecific [43], which ascribes the small number of characterized AMF species (~300) compared to that of plant species (~300,000) [44]. Nevertheless, evidence exists that a preference of AMF‐plant associations was present in different ecosystems [13, 14, 44]. Apart from the release of carbonaceous root exudates triggering the most preferred symbiont [45], soil conditions have been acknowledged to modify the ability of plants to attract selected AMF [46].

Our results showed that soil conditions had no significant effect on AMF composition. This divergence seems likely due to the strong regulation of the plant host on the rhizosphere species pool via root exudates, thereby masking soil conditions in structuring the soil AMF communities. In line with other studies, it was demonstrated that the host plant exerts a much stronger selectivity for AMF colonization of roots than prevailing soil conditions [13, 47, 48]. This confirms the acknowledged community assembly concept of AMF, whereby the host filter was decisive for AMF assemblage within the plant [7]. On the other hand, the effect of soil conditions on AMF richness and diversity was detected. First, AMF richness and diversity correlated negatively with soil pH, as it was observed in other situations [8, 16, 49]. Soil pH controls AMF richness and diversity through influencing nutrient and ion availability [50]. Consequently, we found that the content of soil zinc (Zn) correlated with AMF richness but not diversity, while soil calcium (Ca) correlated with both AMF richness and diversity. Both nutrients facilitate plant metabolic processes, and their uptake is supported by AMF [51]. It is known that high Zn soil levels can negatively affect the abundance and composition of AMF in polluted soils [52, 53], while other studies have shown that Zn could influence AMF diversity and richness in non‐polluted ecosystems [8, 16, 54]. These inconsistent results were most likely explained by differences in ecosystem type and structure. On the other hand, only limited information is available about the influence of Ca on AMF diversity and composition. Ca is not only a nutrient, but also considered as a messenger which initializes the communication between plants and AMF [55]. Further research would be needed to explore the influence of Ca on AMF diversity and composition under the given environmental conditions of our study. Although pH, Zn and Ca had a significant effect on richness and/or diversity of AMF in our study, they did not trigger a significant difference in terms of community assembly.

Our data further supported the concept of host preference, revealing for the first time two different and highly abundant AMF keystone species in two distinct plant compartments: *Rhizophagus* in roots and *Acaulospora* in rhizosphere soils, associated with a single plant species (i.e., *P. phaseoloides*). It was reported that keystone taxa with high abundance have vital contributions for maintaining ecosystem functioning [56]. This may also apply to *Rhizophagus*, which exists in high abundance in the root compartment of *P. phaseoloides. Rhizophagus* is a generally fast growing species (r‐strategy) and, based on its phenotypic traits, was classified as “competitor” in the life history classification system [57]. Thus, *Rhizophagus* may have a competitive advantage to occupy efficiently the root niche with immediate access to plant‐derived resources. Furthermore, plants prefer to deliver more carbon to beneficial symbionts [21, 22], like *Rhizophagus*, which may help plants to be successful in the harsh environmental conditions of abandoned mining sites. With regard to the colonization of degraded ecosystems (e.g., abandoned mining sites), it was proposed that so‐called “founder AMF” species might benefit from this plant‐derived carbon to colonize the plant roots in the early stage of ecological succession [58]. Accordingly, this ecological advantage would outcompete so‐called “AMF latecomers”, benefiting the proliferation of *Rhizophagus* through the soil via colonization of newly formed roots. More importantly, *Rhizophagus* has been recognized as a dominant AMF species that supports plants in the early stages of growth development in agricultural system [41, 59]. This might also be true for degraded ecosystems. In fact, *Rhizophagus* was considered as a prominently abundant taxon, since it has been found in diverse host species and environments (Supporting Information: Table S1). However, a phylogenetic meta‐analysis of most abundant AMF taxa across different ecosystems, such as *Rhizophagus*, indicated that they do not necessarily have the same phylogenetic structure [60]. On the other hand, *Acaulospora* is a slow growing species (k‐strategy) and the trait‐based framework presented *Acaulospora* as “stress‐tolerant” AMF [57]. However, stress‐tolerant AMF were believed to provide a delayed benefit to their host, which is accompanied by an excessive carbon demand from their host [57]. Therefore, our study suggested that the abundant *Rhizophagus* in the roots of *P. phaseoloides* was the result of a good functional match between both partners in this degraded ecosystem.

## CONCLUSION

Our results provided fundamental genetic insights into AMF communities associated with the pioneering plant *P. phaseoloides* colonizing abandoned mining sites in Ghana. Our study showed that geographic and prevailing soil conditions only exerted significant effects on AMF richness and diversity, but not on AMF community composition. Instead, the plant compartment largely explained the differences in AMF composition, with two different functional species in two distinct plant compartments (i.e., *Acaulospora* in rhizosphere soil; *Rhizophagus* in root). This implied that *P. phaseoloides* has a strong selectivity for AMF species, irrespective of soil conditions, emphasizing the ecological plasticity of the host in selecting AMF. The present study was based on a one‐time point sampling. Hence, to fully understand the ecological effects of AMF communities in degraded ecosystems, further studies, including a broader range of abandoned mining sites with distinct environmental conditions and considering multiple AMF proxies (i.e., spore density, intraradical and extraradical hyphae) across various seasons, would provide a more profound insight into the plasticity and responsiveness of AMF compartmentation in association with *P. phaseoloides*, as a suitable ecological basis for restoration of degraded ecosystems.

## METHODS

### Site description and sampling

**Figure 3:**
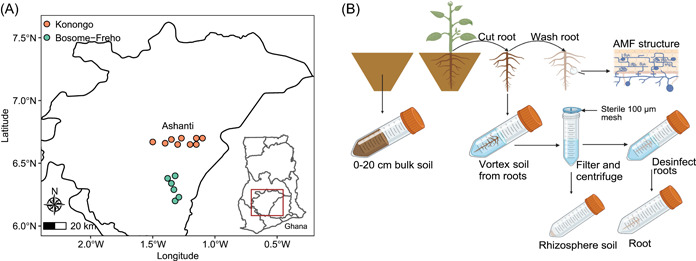
Sampling locations and sampling methods. (A) Sampling sites across two areas within the Ashanti region in Ghana. (B) Sampling methods to collect rhizosphere and root samples.

Soil and root samples were collected at five abandoned gold mining sites distributed across two locations (45 km distance) in the Ashanti region (Ghana) in October 2019. Three sites were located in Konongo (KN, 6°37′ N, 1°14′ E) and two sites in Bosome‐Freho (BF, 6°25′ N, 1°18′ E) (Figure 3A). At each location, all collecting sites had a distance of at least 50 m between each other. Both locations had similar climate conditions (Supporting Information: Table S7), but differed in physic‐chemical soil characteristics (Supporting Information: Table S8). At each site, three spatially separated plant individuals of *P. phaseoloides* with a distance of 1.5 m from each other were randomly selected to ensure independence of samples [54]. The entire plants with soil (approximately 10 cm width and 20 cm depth) were excavated and put into poly bags and transported in cooling boxes. Intact root systems (root balls) were conserved at 4°C [61], permitting samples to be in a semi‐natural state before shipping to Germany (University of Hohenheim, Stuttgart). Upon arrival, samples were conserved at 4°C for further processing.

### Processing of samples

The samples were processed according to the protocol [62], with minor modifications (Figure 3B). Bulk soil was removed from roots by soft shaking and subjected to physic‐chemical analysis (Supporting Information: Table S8). Then, 10–12 roots per plant with a length of 5–8 cm were excised. Excised roots were washed with 35 ml autoclaved, phosphate buffer mixed with 200 g L^−1^ Tween‐20 to detach the rhizosphere soil from roots. The roots were transferred to a new Falcon tube (50 ml) following disinfection procedures: (1) root samples were treated with 35 ml of 50% bleach mixed with 0.01% Tween‐20 for 1 min; (2) the liquid phase was replaced by 35 ml of 70% ethanol for 1 min; (3) root samples were rinsed 5 times with sterile water; (4) washed roots were dried on sterile filter paper. Then, roots were cut into small pieces using sterile forceps and pruning scissors, and conserved at −20°C for DNA extraction. Tubes containing the rhizosphere soils were processed as follows: (1) samples were filtered (sterile 100 µm mesh cell strainer) into a new 50 ml tube; (2) samples were centrifuged (3000*g*, 5 min, room temperature [RT]) and supernatant was removed; (3) tubes were chilled on ice and 1.5 ml of sterile phosphate buffer was added, followed by vortexing; (4) the suspended phase was transferred into a clean 2 ml tube and samples were centrifuged (15,871*g*, 2 min, RT). Finally, the supernatant was removed and rhizosphere soil pellets were conserved at −20°C for DNA extraction.

### Amplicon sequencing

For DNA extraction, 0.1 g frozen roots of each sample homogenized in liquid N, and 0.5 g of frozen rhizosphere soil were used. DNA was extracted with the Fast DNA® spin plant kit (MP Biomedicals). To improve the quantity and quality of rhizosphere soil DNA, 30 mg polyvinylpolypyrrolidone (Thermo Fisher Scientific) was added before buffer addition [63]. DNA concentration was measured by a NanoDrop spectrophotometer 2000 (Thermo Fisher Scientific). Working solutions of root (10‐fold dilution) and rhizosphere soil (5 ng µl^−1^) DNA were prepared with double‐distilled water, and then stored at −20°C for subsequent analysis. Amplicons of *Glomeromycota* were produced with nested PCR. The first PCR used the primers NS31 [64] and AMDGR [65]. Each PCR (20 µl) comprised 1 µl DNA working solution, 0.2 µM of each primer, 0.2 mM of each deoxynucleoside triphosphate (dNTP), 1.5 mM MgCl_2_ and 1 U Taq DNA polymerase (Promega GmbH). Reactions were run on a PeQSTAR thermal cycler (VWR International GmbH, Bruchsal, Germany) using the following conditions: 3 min initial denaturation at 95°C, followed by 35 cycles of 95°C for 30 s, 56° for 30 s, and 72°C for 30 s. Reactions were completed with 72°C for 3 min. The second nested PCR was conducted with the AMF‐specific primers AMV4.5NF and AMDGR [65] tagged with Illumina adapters, yielding an amplicon of approximately 300 bp. One µl of 1:10 diluted amplicon of the first PCR was used for the second PCR in 30 µl reactions, applying the same PCR run conditions as mentioned above, except that the annealing time was reduced to 20 s. Amplicons were verified by a 1.5% agarose gel electrophoresis. Then, 25 µl of amplicons with Illumina adaptors were submitted to Eurofins Genomics Europe Sequencing GmbH. Library construction and quality check were done by Eurofins Genomics. Illumina MiSeq was used for sequencing with a 2 × 300 sequence mode (Eurofins Genomics). The raw sequences were deposited in the Genome Sequence Archive [66] under BioProject accession number PRJ011089.

### Bioinformatic analysis

Sequences were trimmed to exclude primer sequences and quality‐filtered with quality scores > 35 in an initial step [18]. Rare amplicon sequence variants (ASV), with a frequency of less than 0.1% of the mean sequence depth, were removed. As Illumina reported to be 0.1% of reads most likely due to MiSeq bleed‐through between runs (https://github.com/LangilleLab/microbiome_helper/wiki/Amplicon-SOP-v2-(qiime2-2020.2)).

Taxonomic identification of each ASV was performed according to the protocol of Stefani with minor modification [18]. First, each ASV was identified with the closest sequences against the National Center for Biotechnology Information (NCBI), using a basic local alignment search tool (BLAST). The search was set to *Glomeromycotina* (taxid:214504), whereby uncultured/environmental sample sequences were excluded and the maximum number of similar sequences retrieved (i.e., the number of top hits to record) was set to 10. BLAST results were exported as a single file XML2 and uploaded to Geneious Prime® (version 2020.2), with the aim of downloading the taxonomic information and saving only the first hit (the hit with the highest pairwise similarity and query coverage of >95%). Then, BLAST results with taxonomic information were imported to QIIME2. Using qiime feature‐classifier (classify‐consensus‐blast) to assign ASV, sequences belonging to ASV identified as non‐*Glomeromycotina* (unclassified AMF) at the phylum level were removed. The remaining ASV were considered as effective ASV and used for downstream analysis. Taxonomy assignment was inferred with RAxML (v8.2.12) under the GTRGAMMA model and 1000 bootstraps via the CIPRES web‐portal using “phylogenetic backbone tree”. Phylogenetic backbone tree was calculated with the same producer as above, in addition to specify outgroups. The reference sequences were acquired from database [67] and recently described AMF species in public repositories. The taxonomy of each ASV was delimitated with its position in the phylogenetic tree (Supporting Information: Table S9).

### Statistical analysis

All statistical analyses were done in R (version 4.0.3). All sequence information is given in Supporting Information: Table S2. Low sequencing depth samples (<1000) were removed from the analysis to avoid any contamination with poor quality sequences [68] (colored red in Supporting Information: Table S2). Data normality and homogeneity of variance were considered, and *α* = 0.05 was defined as statistical significance. If needed, *p* values were adjusted for multiple comparisons using the Benjamini‐Hochberg method [69].

Rarefaction curve was assembled individually with ASV of each compartment to confirm the sequencing depth. To eliminate errors, samples were rarefied to 1500 sequences before calculating diversity indices. Alpha (α)‐diversity indices, including observed ASV, ASV evenness (Pielou's evenness), Shannon and InvSimpson diversity, were estimated from rarefied ASV. Two‐way analysis of variance was used to test the significant difference in α‐diversity indices within plant compartments and between locations. Post‐hoc comparisons were conducted with Tukey's honest analysis.

Beta (β)‐diversity of AMF communities was calculated using weighted UniFric nonmetric dimensional scaling (NMDS) ordination at the ASV level. Permutational MANOVA was carried out using Vegan's function adonis() to measure significant effects of locations and plant compartment on β‐diversity. To identify distinct ASV of the two compartments, DeSeq2 was performed [70]. Furthermore, to verify whether this difference is related to functional variation among AMF, keystone taxa were determined in both plant compartments (i.e., rhizosphere soil, root). This analysis step was justified since keystone taxa are non‐replaceable in microbiome structure and play a critical role in microbiome functioning [71]. AMF keystone taxa were identified with co‐occurrence networks which were considered as a powerful tool for inferring keystone taxa from microbial communities [71]. Co‐occurrence networks analysis was done in R, using the Spearman correlation coefficient. According to strong (*R* > 0.6) and significant correlation (*p*_FDR_ < 0.05), co‐occurrence models within the rhizosphere soil and root compartment were constructed. Co‐occurrence networks were visualized on the Gephi platform (version 0.9.2) using the Fruchtermann–Feingold layout [72]. Those ASV with the highest betweenness centrality scores were considered as keystone taxa [73].

To detect the relationships between α‐diversity of AMF (observed ASV richness and Shannon diversity) and soil characteristics, Pearson correlation was calculated. To further check the influence of soil characteristics on AMF composition, distance‐based redundancy analysis (db‐RDA) was conducted. The variance inflation factor (VIF) was calculated to select decisive soil characteristics, whereby soil characteristics with VIF values less than 10 were selected [74]. The stepwise db‐RDA was performed with Vegan's function dbrda() in R. The significance of variations in AMF composition explained by soil characteristics was tested by Monte Carlo permutation testing.

## AUTHOR CONTRIBUTIONS

Yaqin Guo and Frank Rasche conceptualized the research program and design of the study. Yaqin Guo and Beloved Mensah Dzomeku collected samples. Yaqin Guo performed the sample analysis in the lab. Yaqin Guo and Qicheng Bei conducted data analysis. Yaqin Guo wrote the manuscript and Konrad Martin edited the manuscript. All authors have commented and approved the final manuscript.

## CONFLICT OF INTEREST

The authors declare no conflict of interest.

## Supporting information

Supporting information.

Supporting information.

## Data Availability

The raw sequences were archived in GSA (PRJ011089) and NCBI Sequence Read Archive (PRJNA739689). The scripts (QIIME2 and R) generated during the current study are available at https://github.com/yaqinguo/AMF-communities-of-the-pioneer-plant-Pueraria-phaseoloides. Supporting Information (figures, tables, scripts, graphical abstract, slides, videos, Chinese translated version and update materials) may be found in the online DOI or iMeta Science http://www.imeta.science/.

## References

[1] Mahmoudi, Neji , Cristina Cruz , Mosbah Mahdhi , Mohamed Mars , and Maria F. Caeiro . 2019. “Arbuscular Mycorrhizal Fungi in Soil, Roots and Rhizosphere of *Medicago truncatula*: Diversity and Heterogeneity Under Semi‐Arid Conditions.” PeerJ 7: e6401. 10.7717/peerj.6401 30842895 PMC6398376

[2] Lee, Eun‐Hwa , Ju‐Kyeong Eo , Kang‐Hyeon Ka , and Ahn‐Heum Eom . 2013. “Diversity of Arbuscular Mycorrhizal Fungi and Their Roles in Ecosystems.” Mycobiology 41: 121–25. 10.5941/MYCO.2013.41.3.121 24198665 PMC3817225

[3] Saia, Sergio , and Jan Jansa . 2022. “Editorial: Arbuscular Mycorrhizal Fungi: The Bridge Between Plants, Soils, and Humans.” Frontiers in Plant Science 13: 875958. 10.3389/fpls.2022.875958 35444670 PMC9014169

[4] Powell, Jeff R. , and Matthias C. Rillig . 2018. “Biodiversity of Arbuscular Mycorrhizal Fungi and Ecosystem Function.” New Phytologist 220: 1059–75. 10.1111/nph.15119 29603232

[5] Stürmer, Sidney L. , James D. Bever , and Joseph B. Morton . 2018. “Biogeography of Arbuscular Mycorrhizal Fungi (Glomeromycota): A Phylogenetic Perspective On Species Distribution Patterns.” Mycorrhiza 28: 587–603. 10.1007/s00572-018-0864-6 30187122

[6] Jansa, Jan , Angela Erb , Hans‐Rudolf Oberholzer , Petr Šmilauer , and Simon Egli . 2014. “Soil and Geography Are More Important Determinants of Indigenous Arbuscular Mycorrhizal Communities Than Management Practices in Swiss Agricultural Soils.” Molecular Ecology 23: 2118–35. 10.1111/mec.12706 24611988

[7] Vályi, Kriszta , Ulfah Mardhiah , Matthias C. Rillig , and Stefan Hempel . 2016. “Community Assembly and Coexistence in Communities of Arbuscular Mycorrhizal Fungi.” Isme Journal 10: 2341–51. 10.1038/ismej.2016.46 27093046 PMC5030697

[8] Xu, Xihui , Chen Chen , Zhou Zhang , Zehua Sun , Yahua Chen , Jiandong Jiang , and Zhenguo Shen . 2017. “The Influence of Environmental Factors on Communities of Arbuscular Mycorrhizal Fungi Associated With Chenopodium Ambrosioides Revealed by MiSeq Sequencing Investigation.” Scientific Reports 7: 45134. 10.1038/srep45134 28327631 PMC5361092

[9] Li, Xiaoliang , Zhiqiang Qi , Xiaolan Yu , Meng Xu , Zhaohua Liu , Gongfu Du , and Yan Yang . 2021. “Soil pH Drives the Phylogenetic Clustering of the Arbuscular Mycorrhizal Fungal Community Across Subtropical and Tropical Pepper Fields of China.” Applied Soil Ecology 165: 103978. 10.1016/j.apsoil.2021.103978

[10] Cheng, Ying , Keiko Ishimoto , Yuko Kuriyama , Mitsuru Osaki , and Tatsuhiro Ezawa . 2013. “Ninety‐Year‐, but Not Single, Application of Phosphorus Fertilizer Has a Major Impact on Arbuscular Mycorrhizal Fungal Communities.” Plant and Soil 365: 397–407. 10.1007/s11104-012-1398-x

[11] Moebius‐Clune, Daniel J. , Bianca N. Moebius‐Clune , Harold M. van Es , and Teresa E. Pawlowska . 2013. “Arbuscular Mycorrhizal Fungi Associated With a Single Agronomic Plant Host Across the Landscape: Community Differentiation Along a Soil Textural Gradient.” Soil Biology and Biochemistry 64: 191–99. 10.1016/j.soilbio.2012.12.014

[12] Torrecillas, E. , M. M. Alguacil , and A. Roldán . 2012. “Host Preferences of Arbuscular Mycorrhizal Fungi Colonizing Annual Herbaceous Plant Species in Semiarid Mediterranean Prairies.” Applied and Environmental Microbiology 78: 6180–86. 10.1128/AEM.01287-12 22752164 PMC3416610

[13] Deepika, Sharma , and David Kothamasi . 2021. “Plant Hosts May Influence Arbuscular Mycorrhizal Fungal Community Composition in Mangrove Estuaries.” Mycorrhiza 31: 699–711. 10.1007/s00572-021-01049-y 34477968

[14] Sepp, Siim‐Kaarel , John Davison , Teele Jairus , Martti Vasar , Mari Moora , Martin Zobel , and Maarja Öpik . 2019. “Non‐Random Association Patterns in a Plant–Mycorrhizal Fungal Network Reveal Host–Symbiont Specificity.” Molecular Ecology 28: 365–78. 10.1111/mec.14924 30403423

[15] Saks, Ülle , John Davison , Maarja Öpik , Martti Vasar , Mari Moora , and Martin Zobel . 2014. “Root‐Colonizing and Soil‐Borne Communities of Arbuscular Mycorrhizal Fungi in a Temperate Forest Understorey.” Botany 92: 277–85. 10.1139/cjb-2013-0058

[16] Alguacil, Maria del Mar , Maria Pilar Torres , Alicia Montesinos‐Navarro , and Antonio Roldan . 2016. “Soil Characteristics Driving Arbuscular Mycorrhizal Fungal Communities in Semiarid Mediterranean Soils.” Applied and Environmental Microbiology 82: 3348–56. 10.1128/AEM.03982-15 27016567 PMC4959246

[17] Coleman‐Derr, Devin , Damaris Desgarennes , Citlali Fonseca‐Garcia , Stephen Gross , Scott Clingenpeel , Tanja Woyke , Gretchen North , et al. 2016. “Plant Compartment and Biogeography Affect Microbiome Composition in Cultivated and Native Agave Species.” New Phytologist 209: 798–811. 10.1111/nph.13697 26467257 PMC5057366

[18] Stefani, Franck , Karima Bencherif , Stéphanie Sabourin , Anissa Lounès Hadj‐Sahraoui , Claudia Banchini Sylvie Séguin , and Yolande Dalpé . 2020. “Taxonomic Assignment of Arbuscular Mycorrhizal Fungi in an 18S Metagenomic Dataset: A Case Study With Saltcedar (*Tamarix aphylla*).” Mycorrhiza 30: 243–55. 10.1007/s00572-020-00946-y 32180012

[19] Maherali, Hafiz , and N. Klironomos John . 2007. “Influence of Phylogeny on Fungal Community Assembly and Ecosystem Functioning.” Science 316: 1746–48. 10.1126/science.1143082 17588930

[20] Babalola, Busayo Jing , Joshua Li , Claire Elizabeth Willing , Yong Zheng , Yong‐Long Wang , Hui‐Yun Gan , Xing‐Chun Li , et al. 2022. “Nitrogen Fertilisation Disrupts the Temporal Dynamics of Arbuscular Mycorrhizal Fungal Hyphae but Not Spore Density and Community Composition In a Wheat Field.” New Phytologist 234: 2057–72. 10.1111/nph.18043 35179789

[21] Liu, Yongjun , Guoxi Shi , Lin Mao , Gang Cheng , Shengjing Jiang , Xiaojun Ma , and Lizhe An , et al. 2012. “Direct and Indirect Influences of 8 yr of Nitrogen and Phosphorus fertilization on Glomeromycota in an Alpine Meadow Ecosystem.” New Phytologist 194: 523–35. 10.1111/j.1469-8137.2012.04050.x 22292929

[22] Stevens, Bo Maxwell , Jeffrey Ryan Propster , Maarja Öpik , Gail W. T. Wilson , Sara Lynne Alloway , Emilian Mayemba , and Nancy Collins Johnson . 2020. “Arbuscular Mycorrhizal Fungi in Roots and Soil Respond Differently to Biotic and Abiotic Factors in the Serengeti.” Mycorrhiza 30: 79–95. 10.1007/s00572-020-00931-5 31970495

[23] Chagnon, Pierre‐Luc , Robert L. Bradley , and John N. Klironomos . 2012. “Using Ecological Network Theory to Evaluate the Causes and Consequences of Arbuscular Mycorrhizal Community Structure.” New Phytologist 194: 307–12. 10.1111/j.1469-8137.2011.04044.x 22269121

[24] Chourasiya, Dipanti , Manju M. Gupta , Sumit Sahni , Fritz Oehl , Richa Agnihotri , Reena Buade , Hemant S. Maheshwari , Anil Prakash , and Mahaveer P. Sharma . 2021. “Unraveling the AM Fungal Community for Understanding its Ecosystem Resilience to Changed Climate in Agroecosystems.” Symbiosis 84: 295–310. 10.1007/s13199-021-00761-9

[25] Douglas, A. Schaefer , Gui Heng , E. Mortimer Peter , and Xu Jianchu . 2021. “Arbuscular Mycorrhiza and Sustainable Agriculture.” Circular Agricultural Systems 1: 1–7. 10.48130/CAS-2021-0006

[26] Asmelash, Fisseha , Tamrat Bekele , and Emiru Birhane . 2016. “The Potential Role of Arbuscular Mycorrhizal Fungi in the Restoration of Degraded Lands.” Frontiers in Microbiology 7: 1095. 10.3389/fmicb.2016.01095 27507960 PMC4960231

[27] Shuab, Razia , Rafiq Lone , Javaid Ahmad , and Zafar A. Reshi . 2017. “RETRACTED CHAPTER: Arbuscular Mycorrhizal Fungi: A Potential Tool for Restoration of Degraded Land.” Mycorrhiza—Nutrient Uptake, Biocontrol, Ecorestoration, Springer International Publishing: 415–34. 10.1007/978-3-319-68867-1_22

[28] Mao, Lin , Jianbin Pan , Shengjing Jiang , Guoxi Shi , Mingsen Qin , Zhiguang Zhao , Qi Zhang , et al. 2019. “Arbuscular Mycorrhizal Fungal Community Recovers Faster Than Plant Community in Historically Disturbed Tibetan Grasslands.” Soil Biology and Biochemistry 134: 131–41. 10.1016/j.soilbio.2019.03.026

[29] Corkidi, L. , and Emmanuel Rincón . 1997. “Arbuscular Mycorrhizae in a Tropical Sand Dune Ecosystem on the Gulf of Mexico.” Mycorrhiza 7: 17–23. 10.1007/s005720050158

[30] Nakatsubo, Takayuki , Masami Kaniyu , Nobukazu Nakagoshi , and Takao Horikoshi . 1994. “Distribution of Vesicular‐Arbuscular Mycorrhizae in Plants Growing in a River Floodplain.” Bulletin of Japanese Society of Microbial Ecology 9: 109–17. 10.1264/microbes1986.9.109

[31] Fujiyoshi, Masaaki , Atsushi Kagawa , Takayuki Nakatsubo , and Takehiro Masuzawa . 2005. “Successional Changes in Mycorrhizal Type in the Pioneer Plant Communities of a Subalpine Volcanic Desert on Mt. Fuji, Japan.” Polar Bioscience 18: 60–72. 10.15094/00006226

[32] Quoreshi, Ali M. 2008. “The Use of Mycorrhizal Biotechnology in Restoration of Disturbed Ecosystem.” In Mycorrhizae: Sustainable Agriculture and Forestry, edited by Zaki Anwar Siddiqui , Mohd. Sayeed Akhtar and Kazuyoshi Futai , 303–20. Dordrecht: Springer. 10.1007/978-1-4020-8770-7_13

[33] Council, World Gold. 9 June 2022, date last accessed. Top ten Gold producing Countries in 2020. https://www.gold.org/goldhub/data/gold-production-by-country?gclid=CjwKCAjwtIaVBhBkEiwAsr7-c2O3lJh_zO9a461Yxvqe0b_4I50cB9wOIZCjoPG_VlddRS4kJls58xoCYL8QAvD_BwE

[34] Barenblitt, Amanda , Abigail Payton , David Lagomasino , Lola Fatoyinbo , Kofi Asare , Kenneth Aidoo , Hugo Pigott , et al. 2021. “The Large Footprint of Small‐Scale Artisanal Gold Mining in Ghana.” Science of the Total Environment 781: 146644. 10.1016/j.scitotenv.2021.146644 33812105

[35] Singhal, Raj K. 2009. “Mining and the Environment: From Ore to Metal.” International Journal of Mining, Reclamation and Environment 23: 241. 10.1080/17480930903429794

[36] Waidyanatha, U. P. de S. , N. Yogaratnam , and W. A. Ariyaratne . 1979. “Mycorrhizal Infection on Growth and Nitrogen Fixation of Pueraria and Stylosanthes and Uptake of Phosphorus From Two Rock Phosphates.” New Phytologist 82: 147–52. 10.1111/j.1469-8137.1979.tb07569.x

[37] Wu, Songlin , Fang You , Zhaoxiang Wu , Philip Bond , Merinda Hall , and Longbin Huang . 2020. “Molecular Diversity of Arbuscular Mycorrhizal Fungal Communities Across the Gradient of Alkaline Fe Ore Tailings, Revegetated Waste Rock to Natural Soil Sites.” Environmental Science and Pollution Research 27: 11968–79. 10.1007/s11356-020-07780-x 31983001

[38] Clarke, K. R. 1993. “Non‐Parametric Multivariate Analyses of Changes in Community Structure.” Australian Journal of Ecology 18: 117–43. 10.1111/j.1442-9993.1993.tb00438.x

[39] Newman, M. E. J. 2006. “Modularity and community structure in networks.” *Proceedings of the National Academy of Sciences of the United States of America* 103: 8577–82. 10.1073/pnas.0601602103 PMC1482622 16723398

[40] Davison, John , Maarja Öpik , Tim J. Daniell , Mari Moora , and Martin Zobel . 2011. “Arbuscular Mycorrhizal Fungal Communities in Plant Roots Are Not Random Assemblages.” FEMS Microbiology Ecology 78: 103–15. 10.1111/j.1574-6941.2011.01103.x 21457278

[41] Gao, Cheng , Liliam Montoya , Ling Xu , Mary Madera , Joy Hollingsworth , Elizabeth Purdom , Robert B. Hutmacher , et al. 2019. “Strong Succession in Arbuscular Mycorrhizal Fungal Communities.” The ISME Journal 13: 214–26. 10.1038/s41396-018-0264-0 30171254 PMC6298956

[42] Willing, Claire E. , Grady Pierroz , Aidee Guzman , Leander D. L. Anderegg , Cheng Gao , Devin Coleman‐Derr , John W. Taylor , Tom D. Bruns , and Todd E. Dawson . 2021. “Keep Your Friends Close: Host Compartmentalisation of Microbial Communities Facilitates Decoupling From Effects of Habitat Fragmentation.” Ecology Letters 24: 2674–86. 10.1111/ele.13886 34523223

[43] Smith, Sally E. , and David Read . 2008. Mycorrhizal Symbiosis. 3rd ed. London: Academic Press, 1–9. 10.1016/B978-012370526-6.50002-7

[44] Vályi, Kriszta , Matthias C. Rillig , and Stefan Hempel . 2015. “Land‐Use Intensity and Host Plant Identity Interactively Shape Communities of Arbuscular Mycorrhizal Fungi in Roots of Grassland Plants.” New Phytologist 205: 1577–86. 10.1111/nph.13236 25545193

[45] Bever, James D. , Sarah C. Richardson , Brandy M. Lawrence , Jonathan Holmes , and Maxine Watson . 2009. “Preferential Allocation To Beneficial Symbiont With Spatial Structure Maintains Mycorrhizal Mutualism.” Ecology Letters 12: 13–21. 10.1111/j.1461-0248.2008.01254.x 19019195

[46] Walder, Florian , and Marcel G. A. van der Heijden . 2015. “Regulation of Resource Exchange in the Arbuscular Mycorrhizal Symbiosis.” Nature Plants 1: 15159. 10.1038/nplants.2015.159 27251530

[47] Sandoz, Frédéric Alexandre , Saskia Bindschedler , Benjamin Dauphin , Laurent Farinelli , Jason R. Grant , and Vincent Hervé . 2020. “Biotic and Abiotic Factors Shape Arbuscular Mycorrhizal Fungal Communities Associated With the Roots of the Widespread Fern *Botrychium lunaria* (Ophioglossaceae).” Environmental Microbiology Reports 12: 342–54. 10.1111/1758-2229.12840 32216046

[48] Horn, Sebastian , Tancredi Caruso , Erik Verbruggen , Matthias C. Rillig , and Stefan Hempel . 2014. “Arbuscular Mycorrhizal Fungal Communities Are Phylogenetically Clustered At Small Scales.” The ISME Journal 8: 2231–42. 10.1038/ismej.2014.72 24824667 PMC4992081

[49] Albornoz, Felipe E. , Suzanne Orchard , Rachel J. Standish , Ian A. Dickie , Gary D. Bending , Sally Hilton , Tim Lardner , et al. 2021. “Evidence for Niche Differentiation in the Environmental Responses of Co‐Occurring Mucoromycotinian Fine Root Endophytes and Glomeromycotinian Arbuscular Mycorrhizal Fungi.” Microbial Ecology 81: 864–73. 10.1007/s00248-020-01628-0 33145650

[50] Neina, Dora . 2019. “The Role of Soil pH in Plant Nutrition and Soil Remediation.” Applied and Environmental Soil Science 2019: 5794869. 10.1155/2019/5794869

[51] Watts‐Williams, Stephanie J. , and Sarah E. Gilbert . 2021. “Arbuscular Mycorrhizal Fungi Affect the Concentration and Distribution of Nutrients in the Grain Differently in Barley Compared With Wheat.” Plants, People, Planet 3: 567–77. 10.1002/ppp3.10090

[52] Yang, Yurong , Yingying Song , Henrik V. Scheller , Amit Ghosh , Yihui Ban , Hui Chen , and Ming Tang . 2015. “Community Structure of Arbuscular Mycorrhizal Fungi Associated With *Robinia pseudoacacia* in Uncontaminated and Heavy Metal Contaminated Soils.” Soil Biology and Biochemistry 86: 146–58. 10.1016/j.soilbio.2015.03.018

[53] Zarei, Mehdi , Stephan König , Stefan Hempel , Mojtaba Khayam Nekouei , Gh Savaghebi , and Francois Buscot . 2008. “Community Structure of Arbuscular Mycorrhizal Fungi Associated to Veronica Rechingeri At the Anguran Zinc and Lead Mining Region.” Environmental Pollution 156: 1277–83. 10.1016/j.envpol.2008.03.006 18439736

[54] Alimi, Afolakemi Abibat , Obinna T. Ezeokoli , Rasheed Adeleke , and Annah Moteetee . 2021. “Arbuscular Mycorrhizal Fungal Communities Colonising the Roots of Indigenous Legumes of South Africa As Revealed By High‐Throughput DNA Metabarcoding.” Rhizosphere 19: 100405. 10.1016/j.rhisph.2021.100405

[55] Thor, Kathrin . 2019. “Calcium—Nutrient and Messenger.” Frontiers in Plant Science 10: 440. 10.3389/fpls.2019.00440 31073302 PMC6495005

[56] Shetty, Sudarshan A. , Floor Hugenholtz , Leo Lahti , Hauke Smidt , and Willem M. de Vos . 2017. “Intestinal Microbiome Landscaping: Insight in Community Assemblage and Implications for Microbial Modulation Strategies.” FEMS Microbiology Reviews 41: 182–99. 10.1093/femsre/fuw045 28364729 PMC5399919

[57] Chagnon, Pierre‐Luc , Robert L. Bradley , Hafiz Maherali , and John N. Klironomos . 2013. “A Trait‐Based Framework To Understand Life History of Mycorrhizal Fungi.” Trends in Plant Science 18: 484–91. 10.1016/j.tplants.2013.05.001 23756036

[58] Pierre‐Luc, Chagnon , L. Bradley Robert , and N. Klironomos John . 2012. “Using Ecological Network Theory to Evaluate the Causes and Consequences of Arbuscular Mycorrhizal Community Structure.” The New Phytologist 194: 307–12. https://www.jstor.org/stable/newphytologist.194.2.307 22269121 10.1111/j.1469-8137.2011.04044.x

[59] Gao, Cheng , Pierre‐Emmanuel Courty , Nelle Varoquaux , Benjamin Cole , Liliam Montoya , Ling Xu , Elizabeth Purdom , et al. 2022. “Successional Adaptive Strategies Revealed By Correlating Arbuscular Mycorrhizal Fungal Abundance With Host Plant Gene Expression.” Molecular Ecology 1–14. 10.1111/mec.16343 35000239

[60] Dumbrell, Alex J. , Michaela Nelson , Thorunn Helgason , Calvin Dytham , and Alastair H. Fitter . 2010. “Idiosyncrasy and Overdominance in the Structure of Natural Communities of Arbuscular Mycorrhizal Fungi: Is There a Role for Stochastic Processes? Journal of Ecology 98: 419–28. 10.1111/j.1365-2745.2009.01622.x

[61] Zettler, Lawrence W. , Landy Rajaovelona , Kazutomo Yokoya , Jonathan P. Kendon , Andrew L. Stice , Amanda E. Wood , and Viswambharan Sarasan . 2017. “Techniques for the Collection, Transportation, and Isolation of Orchid Endophytes From Afar: A Case Study From Madagascar.” Botanical Studies 58: 54. 10.1186/s40529-017-0209-3 29185075 PMC5705530

[62] McPherson, Morgan R. , Peng Wang , Ellen L. Marsh , Robert B. Mitchell , and Daniel P. Schachtman . 2018. “Isolation and Analysis of Microbial Communities in Soil, Rhizosphere, and Roots in Perennial Grass Experiments.” JoVE 137: e57932. 10.3791/57932 PMC6126543 30102263

[63] Cheng, Fei , Lin Hou , Keith Woeste , Zhengchun Shang , Xiaobang Peng , Peng Zhao , and Shuoxin Zhang . 2016. “Soil Pretreatment and Fast Cell Lysis for Direct Polymerase Chain Reaction From Forest Soils for Terminal Restriction Fragment Length Polymorphism Analysis of Fungal Communities.” Brazilian Journal of Microbiology 47: 817–27. 10.1016/j.bjm.2016.06.007 27528083 PMC5052337

[64] Simon, Luc , M. Lalonde , and T. D. Bruns . 1992. “Specific Amplification of 18S Fungal Ribosomal Genes From Vesicular‐Arbuscular Endomycorrhizal Fungi Colonizing Roots.” Applied and Environmental Microbiology 58: 291–95. 10.1128/aem.58.1.291-295.1992 1339260 PMC195206

[65] Sato, Kouichi , Masanori Saito Yoshihisa Suyama , and Kazuo Sugawara . 2005. “A New Primer for Discrimination of Arbuscular Mycorrhizal Fungi With Polymerase Chain Reaction‐Denature Gradient Gel Electrophoresis.” Grassland Science 51: 179–81. 10.1111/j.1744-697X.2005.00023.x

[66] Chen, Tingting , Xu Chen , Sisi Zhang , Junwei Zhu , Bixia Tang , Anke Wang , Lili Dong , et al. 2021. “The Genome Sequence Archive Family: Toward Explosive Data Growth and Diverse Data Types.” Genomics, Proteomics & Bioinformatics 19: 578–83. 10.1016/j.gpb.2021.08.001 PMC9039563 34400360

[67] Krüger, Manuela , Claudia Krüger , Christopher Walker , Herbert Stockinger , and Arthur Schüßler . 2012. “Phylogenetic Reference Data for Systematics and Phylotaxonomy of Arbuscular Mycorrhizal Fungi From Phylum To Species Level.” New Phytologist 193: 970–84. 10.1111/j.1469-8137.2011.03962.x 22150759

[68] Weiss, Sophie , Zhenjiang Zech Xu , Shyamal Peddada , Amnon Amir , Kyle Bittinger , Antonio Gonzalez , Catherine Lozupone , et al. 2017. “Normalization and Microbial Differential Abundance Strategies Depend Upon Data Characteristics.” Microbiome 5: 27. 10.1186/s40168-017-0237-y 28253908 PMC5335496

[69] BenjaminiHochberg, Yoav Yosef , and Yosef Hochberg . 1995. “Controlling the False Discovery Rate: A Practical and Powerful Approach To Multiple Testing.” Journal of the Royal Statistical Society: Series B (Methodological) 57: 289–300. 10.1111/j.2517-6161.1995.tb02031.x

[70] Love, Michael I. , Wolfgang Huber , and Simon Anders . 2014. “Moderated Estimation of Fold Change and Dispersion for RNA‐seq Data With DESeq2.” Genome Biology 15: 550. 10.1186/s13059-014-0550-8 25516281 PMC4302049

[71] Banerjee, Samiran , Klaus Schlaeppi , and Marcel G. A. van der Heijden . 2018. “Keystone Taxa As Drivers of Microbiome Structure and Functioning.” Nature Reviews Microbiology 16: 567–76. 10.1038/s41579-018-0024-1 29789680

[72] Bastian, Mathieu , Sebastien Heymann , and Mathieu Jacomy . 2009. “Gephi: An Open Source Software for Exploring and Manipulating Networks.” *Proceedings of the International AAAI Conference on Web and Social Media* 3: 361–62. https://ojs.aaai.org/index.php/ICWSM/article/view/13937

[73] Banerjee, Samiran , Clive A. Kirkby , Dione Schmutter , Andrew Bissett , John A. Kirkegaard , and Alan E. Richardson . 2016. “Network Analysis Reveals Functional Redundancy and Keystone Taxa Amongst Bacterial and Fungal Communities During Organic Matter Decomposition in an Arable Soil.” Soil Biology and Biochemistry 97: 188–98. 10.1016/j.soilbio.2016.03.017

[74] Hadi, Ali S. , and Samprit Chatterjee . 2006. Regression Analysis by Example. Hoboken, New Jersey: John Wiley & Sons. 10.1002/0470055464.fmatter

