# Supplementary material for: Genetic diversity and community composition of arbuscular mycorrhizal fungi associated with root and rhizosphere soil of the pioneer plant Pueraria phaseoloides
Source: Imeta. 2022 Sep 30;1(4):e51. doi: 10.1002/imt2.51 (PMC10989906; doi:10.1002/imt2.51)
Supplement: Supplementary file 1 — Supporting information. [file IMT2-1-e51-s002.docx]

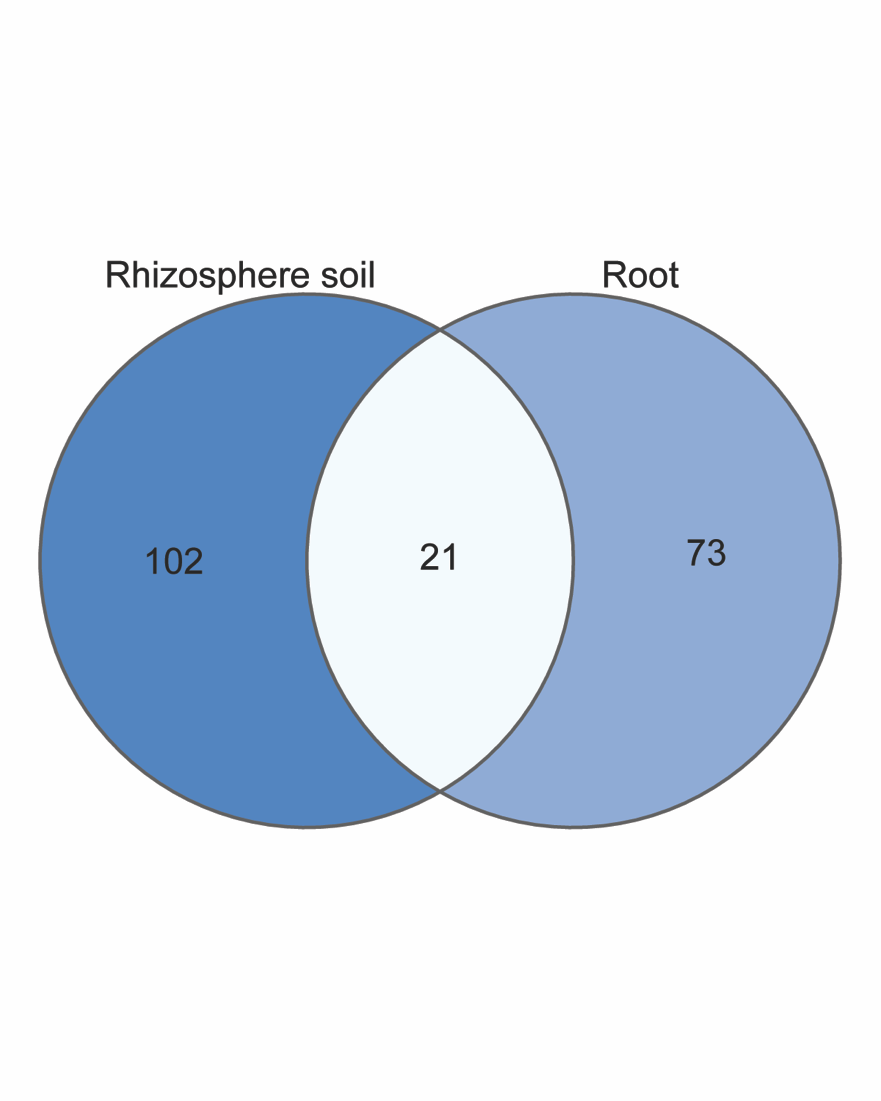


**Figure S1** Venn diagram showing overlap of amplicon sequence variants (ASV) between rhizosphere soil and root compartment. The numbers of ASV were annotated in the figure according to Table S8.


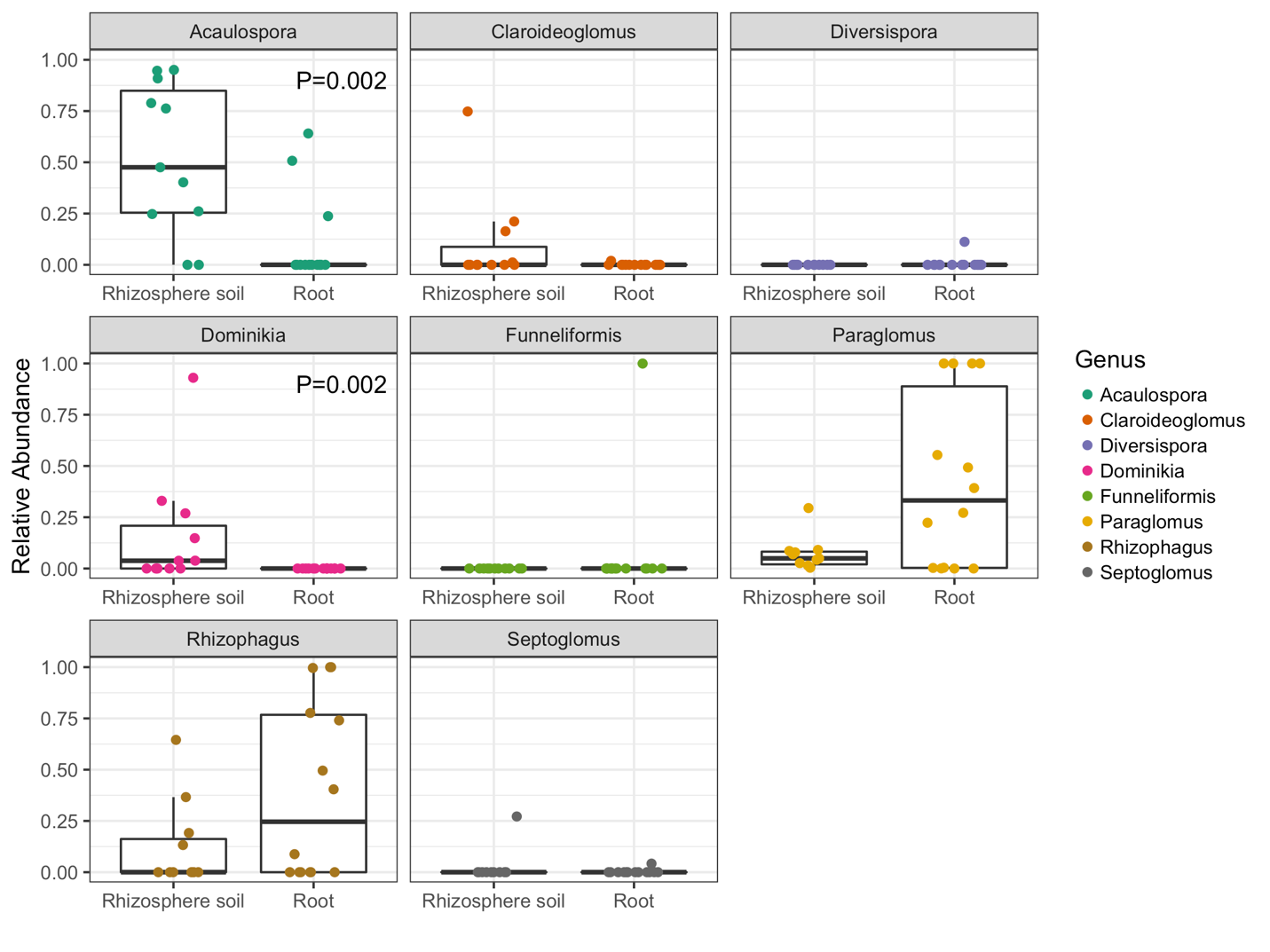


**Figure S2** The relative abundance of each genus in rhizosphere and root compartment with the significant differences between compartments. The relative abundance was calculated based on Figure 1A.


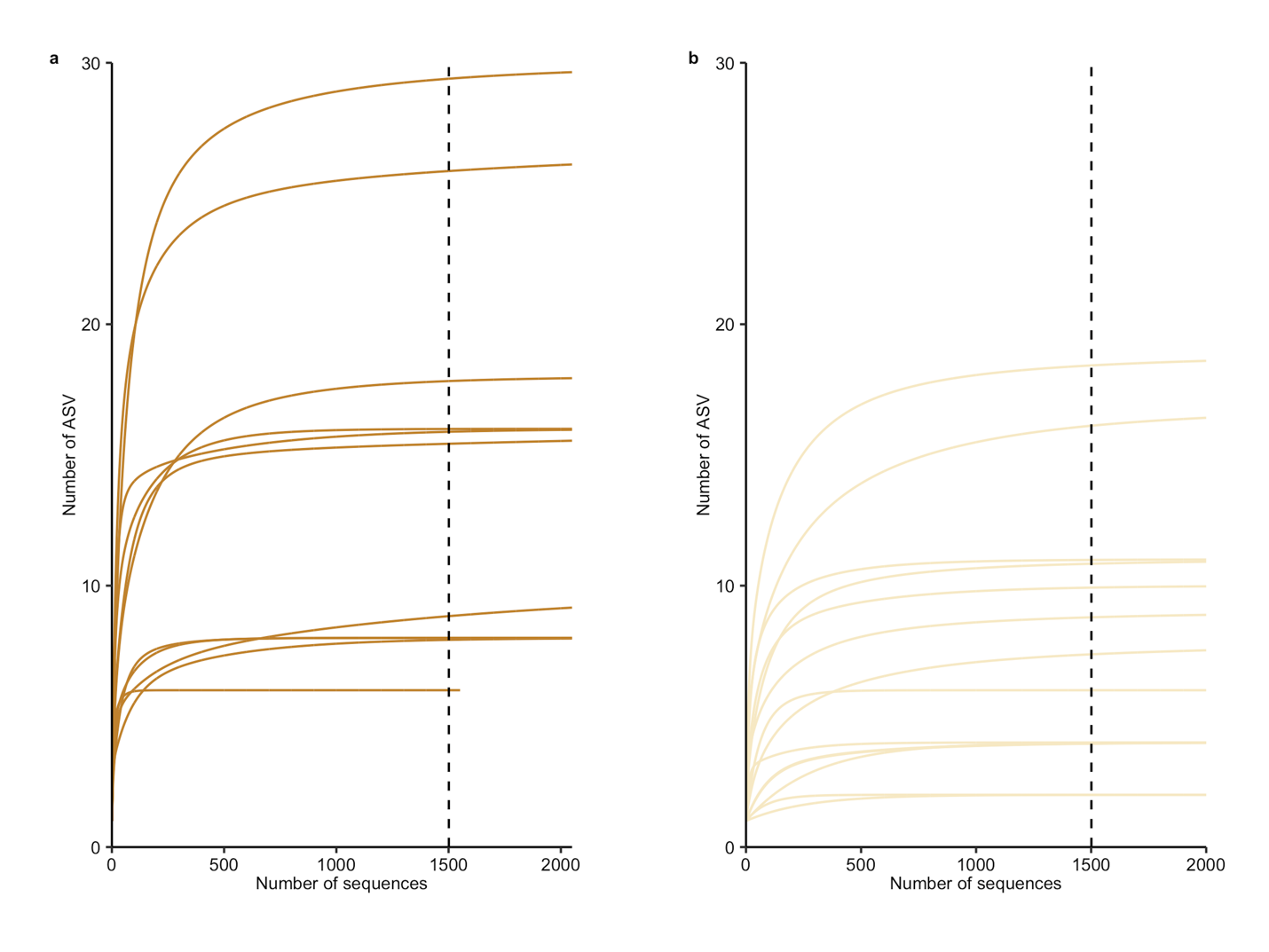


**Figure S3** Rarefaction curves revealing the observed amplicon sequence variants (ASV) in samples from different compartments: a) Rhizosphere soil, b) Root. The black line indicates the minimum sequence of each sample to calculate α-diversity.


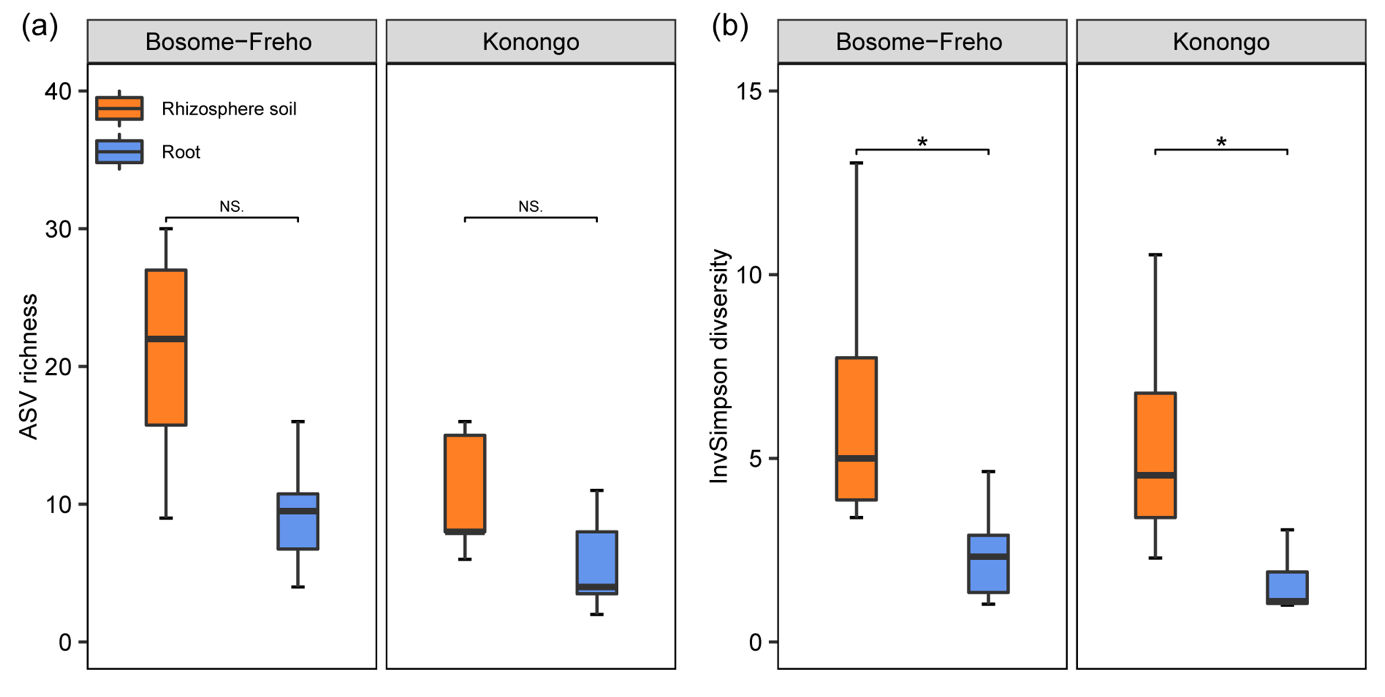


**Figure S4** (a) ASV richness (observed amplicon sequence variants (ASV)). (b) Inverse Simpson diversity. The boxes represent the range between 75^th^ and 25^th^ quartiles. The line within the box represents the median. The whiskers represent the lowest and highest values extending 1.5 of the interquartile range. NS indicates non-significance; * denotes significance (*P<0.05*).
